# Fertile Crescent crop progenitors gained a competitive advantage from large seedlings

**DOI:** 10.1002/ece3.7282

**Published:** 2021-03-04

**Authors:** Catherine Preece, Glynis Jones, Mark Rees, Colin P. Osborne

**Affiliations:** ^1^ Department of Animal and Plant Sciences University of Sheffield Sheffield UK; ^2^ PLECO (Plants and Ecosystems) Department of Biology University of Antwerp Wilrijk Belgium; ^3^ Department of Archaeology University of Sheffield Sheffield UK

**Keywords:** cereal, domestication, Fertile Crescent, functional traits, grass, origins of agriculture

## Abstract

1. Cereal domestication during the transition to agriculture resulted in widespread food production, but why only certain species were domesticated remains unknown. We tested whether seedlings of crop progenitors share functional traits that could give them a competitive advantage within anthropogenic environments, including higher germination, greater seedling survival, faster growth rates, and greater competitive ability.
2. Fifteen wild grass species from the Fertile Crescent were grown individually under controlled conditions to evaluate differences in growth between cereal crop progenitors and other wild species that were never domesticated. Differences in germination, seedling survival, and competitive ability were measured by growing a subset of these species as monocultures and mixtures.
3. Crop progenitors had greater germination success, germinated more quickly and had greater aboveground biomass when grown in competition with other species. There was no evidence of a difference in seedling survival, but seed size was positively correlated with a number of traits, including net assimilation rates, greater germination success, and faster germination under competition. In mixtures, the positive effect of seed mass on germination success and speed of germination was even more beneficial for crop progenitors than for other wild species, suggesting greater fitness. Thus, selection for larger seeded individuals under competition may have been stronger in the crop progenitors.
4. The strong competitive ability of Fertile Crescent cereal crop progenitors, linked to their larger seedling size, represents an important ecological difference between these species and other wild grasses in the region. It is consistent with the hypothesis that competition within plant communities surrounding human settlements, or under early cultivation, benefited progenitor species, favoring their success as crops.

## Graphical abstract

Using controlled‐setting experiments, we studied how the domestication of cereal species in the Fertile Crescent was influenced by the role of seedling competition in species mixtures. Crop progenitors had greater germination success and had greater aboveground biomass than other wild species when grown in competition with other species, and seed size was positively correlated with a number of traits. We provide evidence that the seedlings of cereal crop progenitors have traits that make them better competitors and that interactions with seed size show how they may have amplified their advantage over other wild species during the origins of agriculture.

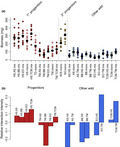

## 1 INTRODUCTION

One of the most significant changes in human behavior began 10,000–12,000 years ago, when people in some regions became progressively more dependent on agriculture rather than hunting and gathering (Barker, 2009; Fuller, 2010; Willcox, 2013). One of the main geographical centers where agriculture began was the Fertile Crescent region in western Asia, where crops including wheat and barley were first domesticated (Abbo et al., 2010; Weiss & Zohary, 2011). However, relatively few species became domesticated as crops, and each center of domestication, including the Fertile Crescent, is home to a large number of other edible species that did not become domesticated. While there is debate over whether or not there was a narrowing in the spectrum of wild plant species collected, and possibly cultivated, over thousands of years (Wallace et al., 2018; Weiss et al., 2004; Willcox et al., 2008), the underlying reasons why some species became domesticated, whereas, others did not remain unknown. Here, we adopt an ecological approach to answering this question, following recent research (Cunniff et al., 2014; Milla & Morente, 2015; Milla et al., 2015; Preece et al., 2015, 2018). We focus on the role of plant traits in the domestication process, through their interaction with the anthropogenic environment including human settlement and early attempts at cultivation, although we acknowledge that there may be other contributing factors, for example, social, economic, and cultural reasons.

Some ecological explanations of crop domestication have considered traits common to crop progenitors that may have given them a competitive advantage in the habitats created when humans first began cultivating the land. One such trait is seed size, as it is well established that cereal crops from the Fertile Crescent and their nearest wild relatives, or progenitors, are amongst the largest‐seeded grasses in the region (Blumler, 1998). This has been more recently demonstrated through direct comparisons between progenitor and non‐progenitor wild species (Kluyver, 2013; Preece et al., 2015). Other traits that are characteristic of Fertile Crescent cereal crop progenitors are to have an annual life‐form, to be monodominant (form monospecific stands) and to have a ruderal growth strategy, thus to be well adapted to disturbed environments (Wood & Lenné, 2018). However, there are many other annual ruderal grasses in that region that were never domesticated. Thus, the mechanisms behind the selection of species, either “unconsciously” by the environment or “consciously” by humans, remain unclear. With this study, we aim to help to fill this knowledge gap, by studying how the domestication of cereal species in the Fertile Crescent was influenced by the role of competition in species mixtures.

Harvested yield is commonly considered an important characteristic of crops and their progenitors (Evans, 1996; Harlan et al., 1973). However, although domesticated cereals of the Fertile Crescent have a greater potential yield than their wild progenitors (Preece et al., 2017), the wild progenitors do not consistently produce a higher yield than other wild grasses available in the region before and during the transition to agriculture (Preece et al., 2015, 2018). Therefore, we propose that characteristics other than grain yield per se may be important in differentiating cereal crop progenitors from other wild grasses. These traits may manifest themselves well before plants reach the mature, seed‐producing stage, and could include characteristics related to germination, seedling survival, and seedling growth. Germination rate and plant growth rate during the establishment phase are both critical for determining competitive outcomes in natural habitats (Martinkova & Honek, 2011), and can vary widely among species. For example, relative growth rates vary by approximately 10‐fold amongst herbaceous species (Poorter & Garnier, 1999) and by fivefold amongst tree seedlings (Grime & Hunt, 1975).

Seedling traits are of particular interest because they have rarely been considered in previous discussions about the origins of agriculture and are related strongly to seed mass, one of the well‐defined traits distinguishing Fertile Crescent cereal crop progenitors from most other wild grass species. Large seed mass has been hypothesized to give seedlings a competitive advantage, for example, when buried deeper in the soil (Purugganan & Fuller, 2009). In support of this hypothesis, comparisons between species show that seed size is positively correlated with the proportions of germinating seeds and seedling survival (Baraloto et al., 2005; Chambers, 1995; Leishman et al., 2000; Metz et al., 2010; Wu & Du, 2007), although evidence for negative relationships also exists (Wu et al., 2011). Seeds also have mechanisms for increasing their chances of germination and survival (and reducing those of their competitors), such as the release of chemical inhibitors, which have allelopathic effects (Friedman & Waller, 1983; Kushima et al., 1998; Zhang et al., 2011), and self‐burying awns to avoid predation (Wood & Lenné, 2018).

The relationship between seed size and plant growth rate is well established, but care must be taken with the metric used, as absolute growth rate (AGR) tends to be higher in larger seeded species (Quero et al., 2008; Reich et al., 1994), and relative growth rate (RGR) (Hunt & Cornelissen, 1997) often declines as plants become larger (Chapin et al., 1989; Maranon & Grubb, 1993; Poorter & Rose, 2005; Swanborough & Westoby, 1996). This issue can be addressed by comparing growth at a common size, which has shown a positive relationship between seed size and growth in short‐lived species (Rees et al., 2010; Rose et al., 2009; Turnbull et al., 2012).

Early cultivation of plants, even prior to the full adoption of agriculture, might have involved management practices such as adding manure, weeding, and watering. A previous study by Cunniff et al. (2014) investigated the functional traits in crop progenitors and wild species to evaluate whether trait differences could explain the selection of progenitors in these early agricultural environments. That work showed that crop progenitors, when grown as individuals up to 4 weeks old, had a number of trait differences from other wild species. This included larger seed and seedling mass, faster germination and higher resilience to defoliation, and that seed size was weakly but positively correlated with growth rate at a common size (Cunniff et al., 2014). This study did implicate seedling RGR as being important in the domestication process, though as it tested a slightly reduced species pool (nine species); it is important to study whether this pattern holds for a larger range of species, as seedling growth is clearly an important component of seedling competitive outcomes.

In this study, we aimed to advance this work by measuring traits in a broader range of species grown as individuals, and by considering the role of intra‐ and interspecific competition. Our work experimentally tested the idea that cereal crop progenitors tend to “win” in the melee of competition in early anthropogenic environments (the fertile and disturbed anthropogenic environments surrounding early human settlements and in early cultivated plots). No study has yet investigated the role of competition in species mixtures in influencing the domestication of cereal species in the Fertile Crescent. The ability of some plants and plant species to out‐compete other plants and species growing in the same human‐mediated environment may potentially account for the natural selection (through habitat filtering) of particular traits, and of certain species as successful crop progenitors. Therefore, greater competitive ability was not necessarily a trait deliberately sought after by early cultivators as, for example, greater seed yield might be.

In order to explore the hypotheses that crop progenitors are both stronger competitors (their growth is greater than other species) and are more tolerant of competition than other species (their growth and yield are less negatively impacted by neighbors), two controlled‐setting experiments were undertaken. We aimed to investigate how traits related to growth, germination, and competition were associated with the selection of crop progenitors, using a set of 15 Fertile Crescent grass species grown in both monocultures and polycultures. The following specific hypotheses were tested: in comparison with other wild grasses, cereal crop progenitors would be as follows: (a) have higher maximum AGR and greater RGR at a particular size; (b) have higher and faster germination rates (in terms of percentage seeds germinated and time to germination) and greater seedling survival; and (c) produce larger plants when grown both under intra‐ and interspecific competition.

## 2 MATERIALS AND METHODS

### 2.1 Plant material

In this study, 15 grass species were selected including the progenitors of the three cereal crop species known to have been domesticated in the south‐western Asian Fertile Crescent region—barley, emmer wheat, and einkorn wheat. These three “primary” crop progenitor species are *Hordeum vulgare* subsp. *spontaneum*, *Triticum monococcum* subsp. *aegilopoides,* and *T. turgidum* subsp. *dicoccoides*. These three species have long been established as primary crop progenitors, with archeobotanical and genetic evidence (Zohary et al., 2012). For the subsequent data analyses, these crop progenitors were compared with nine wild species that were never domesticated (which we call “other wild species”). An additional, three other wild species, *Secale vavilovii* (progenitor of domesticated rye), *Avena sterilis,* and *Avena fatua* (likely progenitors of domesticated oat), which were domesticated but probably at a much later date and outside the Fertile Crescent (Zohary et al., 2012) were also included. To test whether the inclusion of *S. vavilovii, A. sterilis,* and *A. fatua* as “secondary” crop progenitors modified our results, we also performed our analyses with these three additional species, first included as wild species and then as progenitors.

The grass species were selected for experiments because there is archeobotanical evidence that indicates their presence at sites dating to before and during the transition to agriculture or because they are annual species belonging to a genus that is represented at these sites. This was made possible by a database compiled at the University of Sheffield, which collated archeobotanical samples (published and some unpublished) from Epipalaeolithic and Pre‐Pottery Neolithic sites throughout western Asia (Wallace et al., 2018), building on an earlier site‐by‐site database (Connolly & Shennan, 2007). There is wide‐ranging evidence from preserved plant remains, accompanied by evidence of storage and processing of grains, suggesting the importance of annual grasses as a food source at pre‐agricultural and agricultural sites across Southwest Asia (Savard et al., 2006; Weiss et al., 2004; Willcox et al., 2008). For example, evidence for the collection of the species or genera of grasses represented in our study was found at the Upper Palaeolithic site of Ohalo II in Israel (Weiss, Kislev et al., 2004), at Pre‐pottery Neolithic sites in northern Syria (Willcox et al., 2008), and at Neolithic Çatalhöyük in Turkey (Fairbairn et al., 2007).

Seed for the modern representatives of all 15 species was obtained from the Germplasm Research Information Network (GRIN; United States Department of Agriculture; http://www.ars‐grin.gov/aboutgrin.html) (Table S1). While we acknowledge that evolutionary changes will have affected all of the species in the time that has elapsed since early domestication, these modern representatives are the best proxy for the plants that would have been present at that time. Furthermore, we do not expect systematic differences in the strength of selection between progenitors and non‐progenitors. For most species, between two and five accessions per species were used, with only one accession available for three species. The majority of accessions (30 out of 33) were from populations originally collected from south‐western Asia, including the Fertile Crescent and neighboring countries. Accessions were selected to try to maximize variation in individual seed mass within species. This plant material was then used in two separate experiments, hereafter called the “growth experiment” and the “competition experiment”.

### 2.2 Growth experiment

Seeds of the 15 species were weighed individually after removing the outer glumes, and then germinated in trays two‐thirds filled with washed sand (Chelford 52; Sibelco UK Ltd). The sand was saturated with water and 18–26 seeds per species were placed on the surface in rows, enabling individual seedlings to be identified later. Seeds were lightly covered with sand and propagator lids were placed on trays (separated by species), which were then transferred to a controlled environment room (Conviron BDW 40; Conviron). Conditions were maintained at 20/10°C (day/night) with an eight‐hour photoperiod and PPFD of 300 µmol m^−2^ s^−1^. The general method for the growth analysis experiment was grounded in a number of previous studies (Grime & Hunt, 1975; Hunt & Cornelissen, 1997; Poorter, 1989; Poorter & Remkes, 1990).

Two identical growth experiments were established (due to logistical constraints that did not allow one large experiment) with the same experimental setup, but with different accessions used in each experiment (and some variation in species) (Table S2). For each experiment, 12 seedlings per species were randomly selected, three days after germination (from those which had successfully germinated). Seedlings were transferred to one‐liter pots containing washed sand and returned to the controlled environment room with the following conditions: 20/10°C (day/night) with a 16‐hr photoperiod, maximum PPFD of 756 µmol m^−2^ s^−1^. Pots were top‐watered with full strength Long Ashton solution (nitrate version) [(Hewitt, 1966) tables 40, 41] every two days and bottom‐watered with distilled water on alternate days. Seedlings were grown as individuals (one seedling per pot), allowing maximum potential growth rates to be measured (Grime & Hunt, 1975), free from competition.

Six harvests were carried out within a three‐week period, starting on day eight or nine after germination and proceeding every three to four days, finishing on day 27 or 28. Specific timing of harvests varied between experiments one and two, but had no effect on the subsequent growth data. At each harvest, two plants of each species were removed from the pots, washed clean, and divided into roots, leaves, and stems (defined as leaf sheath plus culm). Area of leaves and roots were recorded using a flatbed scanner, and images were later analyzed using Image J (Rasband, W. S., ImageJ, U. S. National Institutes of Health, Bethesda, Maryland, USA, http://imagej.nih.gov/ij/). Plants were dried to a constant weight for three days at 45°C and then dry weight was determined. There were 12 plants per species per experiment, and these were divided between the six harvest points, to make two to four replicates for both experiments (301 pots in total).

Relative growth rate, using the classical approach (ln(final mass/initial mass)/Δtime)), and AGR (final mass − initial mass)/Δtime) were both calculated for the period between the first and final harvests. Growth rate was also calculated by performing a species‐specific functional growth analysis. Growth functions were fitted to plots of logged total plant mass against time, which allowed estimates of RGR at a common size (sRGR) for each species (for full details of the fitting and RGR estimation see: Rees et al., 2010; Rose et al., 2009; Taylor et al., 2010; Turnbull et al., 2012). For this analysis, the common size used was the log of the smallest size common to all species, which was 34.6 mg. Further traits were measured on plants from the final harvest (27 or 28 days after germination). Specific leaf area (SLA) was calculated for each plant as the leaf area (of all leaves) divided by the mass of those leaves (m^2^/kg) and net assimilation rate (NAR) as the increase in dry mass per unit leaf area per unit time (g cm^−2^ d^−1^). Root:shoot ratio was calculated as the mass of roots divided by the mass of the aboveground parts (leaves and stem).

### 2.3 Competition experiment

A subset of nine species used in the growth experiment was subsequently used in a greenhouse competition experiment. The species included the primary and secondary crop progenitors and a reduced set of other wild species (see Table S3). Seeds were weighed individually after removal of outer glumes, and then placed on the substrate surface (1:1 mixture of John Innes no. 2 and sand) in 11‐liter square pots (21 × 21 × 25 cm), arranged in a regular grid pattern with 24 seeds per pot. Seeds of the nine species were planted as monocultures, and then, there were eight combinations of wild‐progenitor in two‐species mixtures (full information in Table S3) with 12 seeds of each species, planted alternately. Mixtures were chosen as a representative sample of the available species, keeping in mind the higher proportion of wild species compared with progenitors (i.e., as south‐western Asian progenitor species are fewer in number than other wild species in the region, we selected two progenitor and four other wild species). There were 10 replicates of each of the monoculture and two‐species combinations, arranged in 10 blocks, making 170 pots in total. Seeds of the different accessions were mixed between the replicates, such that each pot had a roughly equal proportion of the available accessions. The greenhouse chamber conditions were set to 24°C/15°C day/night and a 12‐hr day length, and the growing substrate was kept moist.

Seed germination was checked daily. This provided information on germination success and the time (in days) until germination. Seedling survival was subsequently monitored to know the number of seedlings that were still alive at the end of the experiment. After a period of six weeks since sowing, plant aboveground biomass was harvested and weighed, and calculated as mass per seed sown.

### 2.4 Statistical analyses

All data were analyzed in R (R Core Team, 2016). Data from the growth experiment consisted of AGR, classical RGR (RGR), sRGR, individual seed mass, root:shoot ratio, final leaf area, final root area, SLA, and NAR. AGR, RGR, and sRGR were analyzed using linear models, and other traits were analyzed using linear mixed models with the *lmer* function in in *lme4* (Bates et al., 2015)*,* with species as a random effect. Natural log transformations were applied to all variables apart from classical RGR and sRGR, which are calculated on the basis of log mass. Firstly, the effect of domestication status (crop progenitor or other wild species) on plant traits was tested on its own (as a single fixed effect). Secondly, to understand the role of seed size, natural log‐transformed seed mass was added to the model as a second fixed effect (covariate), along with its interaction with domestication status. We tested for the significance of this interaction, with model selection performed using likelihood ratio tests, with the *lrtest* function in the *lmtest* package (Zeileis & Hothorn, 2002).

Data from the competition experiment included germination success and seedling survival, which were analyzed as binomial variables (family = “binomial”) with *glmer* in the *lme4* package. Block and species were included as random effects. Time to germination and seedling aboveground biomass after six weeks were analyzed with linear mixed effects models using *lmer* in the *lme4* package, with species and block as random effects. The effect size of growing with neighbors of a different species was determined as the relative interaction index (RII), calculated as: RII = (*B*_mi_ − *B*_mo_)/(*B*_mi_ + *B*_mo_), where *B*_mi_ is the biomass of the species growing in a mixture, and *B*_mo_ is the biomass of the species growing as a monoculture (following Armas et al., 2004). This index was calculated both for specific pairs of species, and for species growing in mixtures in general.

As for the growth experiment, each trait was first compared between crop progenitors and other wild species, and then individual seed mass and stand type (monoculture or mixture) was included in the model, including any significant interactions (checked with the *lrtest* function, as before). The functions *lsmeans* and *lstrends* in the *lsmeans* package were used to test for differences between pairs of treatment combinations for time to germination and seedling mass, for example, progenitors in monoculture versus wild species in mixtures. These functions compute least‐squares means for factors in a fitted model or slopes of fitted lines.

All mixed effects models were also checked with a Bayesian approach using the function *MCMCglmm* in the package of the same name (Hadfield, 2010). Differences were only noted for the SLA and NAR data, which are explained in the results.

## 3 RESULTS

### 3.1 Growth experiment

**Figure 1:**
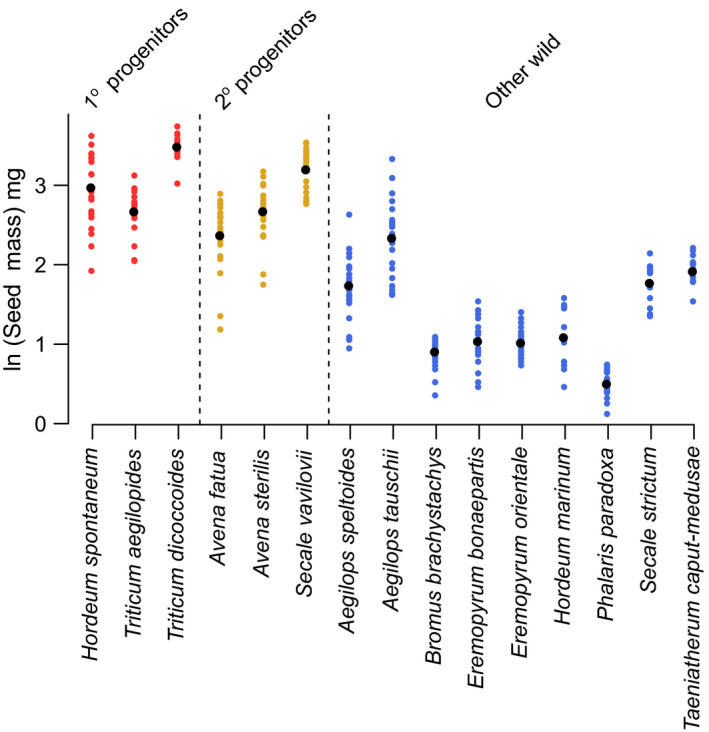
Natural‐logged seed mass for each species (mg). For each species, the small colored points show data for individual replicates while the large black points are the species means. Crop progenitors were significantly larger seeded than other wild species when the secondary crop progenitors were excluded (*p* <.01), and when the secondary progenitors were included as wild species (*p* <.05) and as crop progenitors (*p* <.001)

**Figure 2:**
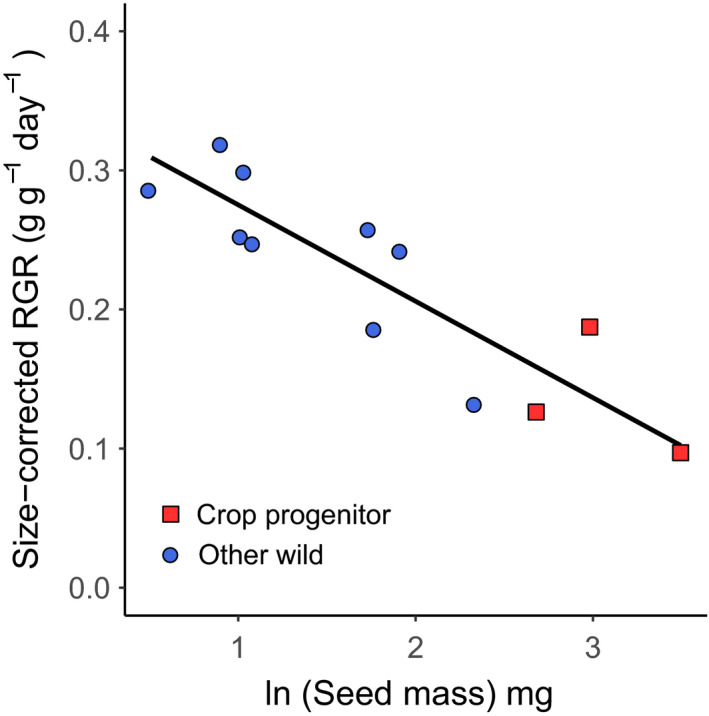
The relationship between size‐corrected relative growth rate and natural log transformed individual seed mass. Each point is a species, with red squares showing primary crop progenitors, and blue circles showing other wild species. Significant effect of seed mass (slope = −0.07, *p* <.001)

The wild progenitors of crops were 5.3 × larger‐seeded than the other wild grasses (Figure 1 and Table 1, linear mixed effects model, *t* = −4.5, *p* <.01), but had lower size‐corrected growth rate (sRGR) than the other grasses (Table 1, linear model, *t* = 3.0, *p* <.05). However, there was no difference in AGR or classical RGR. The effect of domestication status on sRGR was due to the seed mass difference, as when seed mass was included in the model; it was the only significant term (Figure 2 and Table 1, linear model, *t* = −5.9, *p* <.001). There was no effect of initial seed mass on AGR or RGR. When the three secondary crop progenitors were included in the analyses as wild species, the only difference was a positive relationship between AGR and initial seed mass. When they were included as crop progenitors, progenitors also showed higher AGR compared with other wild species (Table 2).

**Table 1:** Effect of domestication status (progenitor vs. wild) and individual seed mass on a range of functional traits

Trait	Progenitors compared with wild species	Effect of logged individual seed mass
*Growth experiment*		
Individual seed mass	×5.3**	N/A
AGR	NS	NS
RGR	NS	NS
sRGR	×0.55*	*** (slope = −0.07)
Root:shoot	NS	NS
Final leaf area	NS	* (slope = 0.43)
Final root area	NS	** (slope = 0.63)
SLA	NS	Interaction with status***
NAR	NS	Interaction with status **,^a^
*Competition experiment*		
Germination success	Progenitors have higher germination success***	3‐way interaction (seed mass × domestication status × stand type)*
Time to germination	NS	3‐way interaction (seed mass × domestication status × stand type)***
Seedling survival	NS	NS
Seedling biomass	× 2.7*	** (slope = 0.61)

Note

Data in the second column show the magnitude of difference between primary progenitors and other wild species. Data in the third column show the effect of logged individual seed mass on the trait, including the size of the slope, when relevant. Three‐way interactions are with status and stand type (monoculture vs. mixture). Asterisks denote significant differences: * = *p* <.05, ** = *p* <.01, *** = *p* <.001. All variables were natural log transformed, except for RGR, sRGR, germination success and seeding survival.

^a^ This interaction was not significant after the removal of a datapoint of the crop progenitors, which had much lower NAR than other crop progenitors.

**Table 2:** Effect of domestication status and individual seed mass on functional traits, with the inclusion of the secondary crop progenitors, *Avena fatua* (only in the growth experiment), *Avena sterilis* and *Secale vavilovii*. These species were included first as wild species and then as progenitors

Trait	Secondary crop progenitors treated as wild		Secondary crop progenitors treated as progenitors	
	Progenitors compared with wild species	Effect of logged individual seed mass	Progenitors compared with wild species	Effect of logged individual seed mass
Seed mass	×3.8*	N/A	×4.6***	N/A
AGR	NS	+ve* (slope = 0.78)	×4.0*	+ve* (slope = 0.78)
RGR	NS	NS	NS	NS
sRGR	×0.60*	−ve*** (slope = −0.07)	×0.63*	−ve*** (slope = −0.07)
Root:shoot	NS	NS	NS	NS
Final leaf area	NS	+ve** (slope = 0.50)	×3.1**	+ve** (slope = 0.50)
Final root area	NS	+ve*** (slope = 0.68)	×3.4**	+ve*** (slope = 0.68)
SLA	NS	interaction with status***	NS	Interaction with status*
NAR	NS	interaction with status*	NS	+ve* (slope = 0.16)
Germination success	NS	3‐way interaction*	Progenitors have higher germination success***	3‐way interaction**
Time to germination	NS	3‐way interaction***	NS	3‐way interaction***
Seedling survival	NS	NS	NS	Interaction with status*
Seedling biomass	NS	+ve*** (slope = 0.70)	× 3.2**	+ve*** (slope = 0.70)

Note

Asterisks denote significant differences: * = *p* <.05, ** = *p* <.01, *** = *p* <.001.

**Figure 3:**
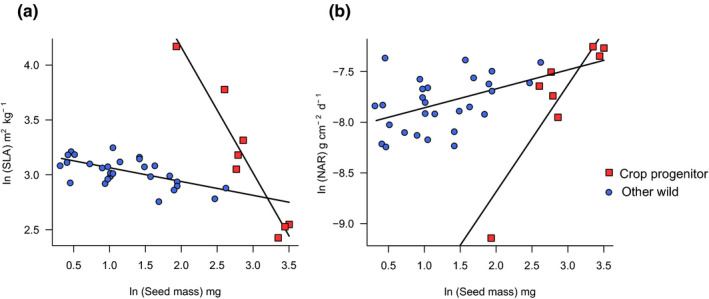
Relationships between (a) specific leaf area (SLA), (b) net assimilation rate (NAR), and individual seed mass in primary crop progenitors and other wild species. Results are plotted from the estimates of a linear mixed effects model with domestication status and ln(seed mass) as fixed effects and species as a random effect. There were significant interactions between domestication status and ln(seed mass) for both SLA (*p* <.001) and NAR (*p* <.01)

There was no effect of domestication status on root:shoot ratio, final leaf or root area, SLA or NAR. Initial seed mass did have significant effects on some traits, such as the positive relationships with final leaf area (linear mixed effects model, *t* = 2.3, *p* <.05), and final root area (linear mixed effects model, *t* = 3.5, *p* <.01). For SLA, there was a significant interaction between domestication status and individual seed mass (linear mixed effects model, *t = *6.5, *p* <.001, (Figure 3a), with the negative correlation between SLA and seed mass being much stronger in crop progenitors. There was also a significant interaction between domestication status and seed mass on NAR (linear mixed effects model, *t* = −3.1, *p* <.01) (Figure 3b), with the positive correlation between NAR and seed mass being much greater in crop progenitors. However, one replicate of the crop progenitors showed a much lower NAR than other crop progenitors, and while this value does fall within the range found in other studies, a leverage plot showed that it exerted a disproportionate influence over results of the model. After the removal of this datapoint, the interaction was no longer significant.

The inclusion of *A. fatua*, *A. sterilis,* and *S. vavilovii* as “other wild species” did not affect any results. When these secondary progenitors were analyzed as additional crop progenitors, leaf and root area were greater in progenitor species, and the domestication status‐seed mass interaction for NAR was replaced by a positive correlation with individual seed mass. Moreover, for both SLA and NAR, regardless of the treatment of the secondary progenitors (omitted, treated as wild, or treated as crop progenitors) when a Bayesian approach was used, the interaction was not detected, indicating that this result must be carefully interpreted and may be strongly influenced by a few individuals in this study.

### 3.2 Competition experiment

**Figure 4:**
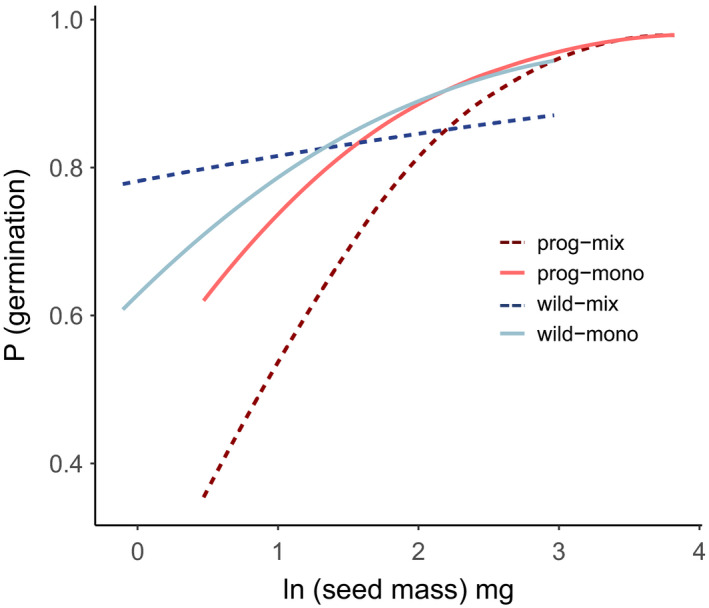
Germination success shown as the proportion of germinating seeds varying with ln(seed mass), domestication status (prog = progenitor) and stand type (mix = mixture, and mono = monoculture). Results are plotted from the estimates of a generalized linear mixed effects model, with a three‐way interaction between seed mass, domestication status, and stand type (*p* <.05). Data are plotted over the range of seed size found for crop progenitors (1.6–45.7 mg) and other wild species (0.9–19.4 mg). In mixtures, seed size increase was more beneficial for crop progenitors (dark red dashed line) compared with wild species (dark blue dashed line)

Crop progenitors had higher germination success than other wild grasses (generalized linear mixed effects model, *z* = −3.59, *p* <.001). There was also a three‐way interaction between seed mass, domestication status, and the stand type (generalized linear mixed effects model, *z* = 1.99, *p* <.05) such that, in mixtures, seed mass has an even greater positive effect for crop progenitors compared with other wild species (Figure 4). Time to germination was not affected by domestication status as a single fixed effect. However, there was a significant three‐way interaction with domestication status, stand type, and individual seed mass (linear mixed effects model, *t* = −5.8, *p* <.001), such that the positive effect of larger seeds was greatest for progenitors in mixtures. Also, time to germination was longer in mixtures than in monocultures (linear mixed effects model, *t* = −6.7, *p* <.001).

The inclusion of *A. sterilis* and *S. vavilovii* in the analysis affected the significance of the single factor effect of domestication status on germination success, with no effect shown when they were included as wild species. Other results relating to germination were unchanged, regardless of whether these two species were treated as wild or progenitors (Table 2).

There were no significant effects of domestication status, stand type, or seed mass on seedling survival. When *A. sterilis* and *S. vavilovii* were included as crop progenitors an interaction was found between seed mass and domestication status, such that survival of wild species decreased with seed mass, while survival of progenitors was unaffected by seed mass. Seedling aboveground biomass (measured after 6 weeks) was higher in crop progenitors (linear mixed effects model, *t* = −3.4, *p* <.05, Table 1 and Figure S1). However, when individual seed mass was included in the model it was the only significant factor (linear mixed effects model, *t* = 5.0, *p* <.01), with larger seeds making larger seedlings (Figure S2).

**Figure 5:**
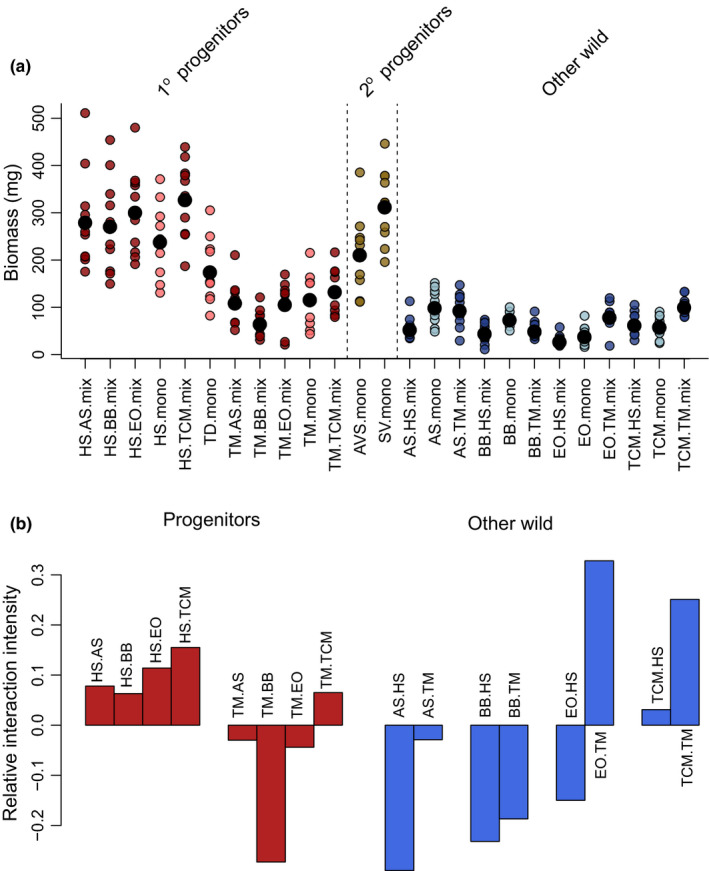
Aboveground biomass of species grown as a monoculture or in a mixture with a second species (a) and the relative interaction index (RII) of growing with a neighbor (b). A positive RII indicates that plants have greater biomass in mixtures than as a monoculture, and vice versa. HS = *H. spontaneum*, T. D = *T. dicoccum* subsp. *dicoccoides*, TM = *T. monococcum* subsp. aegilopoides, AVS = *A. sterilis*, AS = *A. speltoides*, BB = *B. brachystachys*, EO = *E. orientale*, SV = *S. vavilovii*, TCM = *T. caput‐medusae*. For mixtures, the species written first in the code shows the species plotted. For example, “HS.AS” are the data for *H. spontaneum* when grown with *A. speltoides*. In (a), small colored points show replicates and large black circles are the treatment means

Species behaved quite differently from each other, for example, *H. spontaneum* had high biomass (Figure 5a) regardless of when growing in monoculture or mixture, and plants were larger in mixtures than in monocultures when growing with the four wild species (Figure 5b, Table 3). This was not found for the einkorn wheat progenitor (*T. monococcum* subsp. *aegilopoides*), which was negatively affected by growing with three of the four other wild species (Figure 5b, Table 3). Of the wild species, *A. speltoides* and *B. brachystachys* were negatively affected by growing with the crop progenitors, and *E. orientale* was negatively affected by growing with *H. spontaneum* (Figure 5b, Table 3). There was a reciprocal positive effect on biomass between *T. caput‐medusae* and both crop progenitors, such that all three species grew to a larger size in mixture than in monoculture (Figure 5b). Our data indicate that interspecific competition is greater than intraspecific competition for *T. monococcum* subsp. *aegilopoides*, *A. speltoides,* and *B. brachystachys*, but with this relatively small sample size, generalizations about overall competitive ability of crop progenitors versus other wild species cannot be made.

**Table 3:** Overall relative interaction index per species calculated as (biomass in a monoculture subtracted from biomass in a mixture)/(biomass in a monoculture added to biomass in a mixture)

Species	Domestication status	Relative interaction index
*Hordeum vulgare* subsp. *spontaneum*	Progenitor	0.10
*Triticum monococcum* subsp. *aegilopoides*	Progenitor	−0.25
*Aegilops speltoides*	Wild	−0.14
*Bromus brachystachys*	Wild	−0.21
*Eremopyrum orientale*	Wild	0.15
*Taeniatherum caput‐medusae*	Wild	0.15

Note

Positive values indicate that the species produced higher biomass in a mixture compared with a monoculture, and negative values indicate that the species produced lower biomass in a mixture compared with a monoculture.

*Avena sterilis* and *S. vavilovii*, the secondary crop progenitors, had high biomass values which appeared more similar to the primary crop progenitors than to the other wild species. In fact, when these species were analyzed as progenitors, crop progenitors had more than three times greater seedling biomass than wild species (Table 2).

## 4 DISCUSSION

This study evaluates how seedling traits differ between cereal crop progenitors and other wild grasses in order to explore the hypothesis that the competitive advantage of crop progenitors helps to explain why they were domesticated in the Fertile Crescent, while other wild grasses were not. This study is the first to describe the interactions between seed size and neighbor interactions on germination and growth traits during seedling establishment. Although many traits do not differ significantly between crop progenitors and other wild grasses, we show that crop progenitors do have higher germination success and greater seedling biomass. Similar to other studies, we found that individual seed mass has a strong positive influence on growth and competitive ability, and moreover, we demonstrate that the positive impacts of being large‐seeded are greater for crop progenitors, particularly when growing in competition with other wild species.

### 4.1 Seedling growth potential

There were no differences in AGR or RGR between crop progenitors and other wild grasses when grown as individuals, except for an increase in AGR in progenitors, when the secondary crop progenitors were also included in the crop progenitor group. However, growth rate was lower in crop progenitors (0.14 g/g day^−1^ compared with 0.25 g/g day^−1^ for wild) when compared at a common size (sRGR), indicating that these species suffer a growth rate penalty rather than an advantage, when their large seed size is accounted for. This effect was consistent when secondary crop progenitors were included in the analysis (either as progenitors or wild species), indicating that this is a robust result. This result contrasts with an earlier study comparing the traits of three cereal crop progenitors and six wild grass species, which found a weak but significant positive correlation between size‐corrected RGR and seed mass (Cunniff et al., 2014). That study tested a smaller range of species than the current study (there were nine species including the same three primary progenitors and then six other wild species—four of which were used in our study and two congeners of species in this study), and the pattern was predominantly driven by the very high sRGR values for *Hordeum spontaneum*. The fact that we did not replicate this result indicates either that it is not a general pattern over a large sample of species or that there may be a genotype × environment interaction, such that differences in conditions between the different studies strongly influenced the plant traits.

The lack of an advantage in growth rates for crop progenitors is not necessarily surprising because all but one of the species included in this experiment are annuals, and many are ruderal species with adaptations for success in fertile, disturbed environments, including a fast RGR in the seedling phase (Grime, 2001; Wood & Lenné, 2018). In fact, overall, the crop progenitors of barley, emmer wheat, and einkorn wheat have very similar trait combinations to the other wild annual grasses of the region.

The importance of associations between seed size and other functional traits was repeatedly demonstrated in this study, with positive relationships with seed size shown for final leaf area, root area and NAR (suggesting greater net photosynthesis), and negative relationships with sRGR and SLA. The associations with individual seed size seem to be particularly significant for crop progenitors, and relatively less so for other wild species, shown by the interactions between domestication status and seed size for SLA and NAR. While increasing seed mass had strong relationships with decreasing SLA and increasing NAR in crop progenitors, the effects in the same direction were much less pronounced for other wild grasses, pointing to the additional importance that seed size has for crop progenitors. It is important to remember that trade‐offs between seed size and other traits are likely not to be simple, and traits are better understood as being linked together as a suite of interacting characteristics (Moles & Westoby, 2006). Moreover, although this study was concerned primarily with interspecific differences, any increases in seed size within a species may also provoke greater‐than‐average changes in other traits for crop progenitors. These results raise the possibility that the selection for larger seeds might have occurred indirectly due to selection on the associated traits like NAR, or even that all of these traits are under indirect selection via another distinct trait not measured in this study. It is theoretically possible that plants with large seeds were a preferred food source, and that the accompanying competitive advantages were an additional benefit. However, archeobotanical evidence indicates that small‐seeded grasses were an (arguably more) important source of food for pre‐agricultural groups (Savard et al., 2006; Weiss et al., 2004). Furthermore, earlier experiments have shown that large‐seeded grass and legume progenitor species are not necessarily higher yielding than smaller‐seeded species in the same families (Preece et al., 2015, 2018).

### 4.2 Germination success under competition

Crop progenitors had higher germination success than other wild species, and there were positive correlations between germination success, speed of germination, and individual seed mass. This link with seed size is consistent with the majority of previous research into grasses, including work with sea lyme grass (Greipsson & Davy, 1995), durum wheat (Akinci et al., 2008), switchgrass (Aiken & Springer, 1995), and alpine meadow grasses (Wu & Du, 2007). However, note that negative correlations between seed mass and germination rate have been demonstrated in rice (Krishnasamy & Seshu, 1989).

Species with a greater proportion of germinating seeds are likely to have greater seedling success through a higher population density and greater fitness, and are therefore more likely to persist within a habitat (Martinkova & Honek, 2011). Of course, lower germination rates can arise from a greater level of seed dormancy. Seed dormancy is an adaptive trait that can maximize the chance of seeds only germinating in favorable conditions, and can also be a form of “bet‐hedging” in variable environments (Bewley, 1997; Van Klinken et al., 2008). Although longer seed dormancy has historically been predicted to be associated with smaller‐seeded species (Rees, 1994), studies have both supported this theory (Jurado & Flores, 2005; Wu & Du, 2007) and contradicted it (Bu et al., 2007; Norden et al., 2009), showing that this may not be a universal pattern. Long seed dormancy is maladaptive for cultivated species, and crop species therefore tend to germinate soon after planting, with loss of dormancy being part of the domestication syndrome (Larson et al., 2014).

### 4.3 The importance of growing in a mixture or monoculture

Our results for germination success indicate that having large seeds was most beneficial for crop progenitors growing in mixtures with another wild grass and was least beneficial for wild species in mixtures with a crop progenitor. This leads us to consider the importance of the competitive environment that a plant is growing in, and whether the plant is growing in competition with conspecifics in a monoculture or with other species in a mixture. We hypothesize that, when plants were growing as mixtures of different species, larger seeded individuals would have been favored by selection much more strongly in the crop progenitors than in the other wild species because the larger seeds of progenitors had other traits that promoted fitness, such as higher success of seed germination.

Additionally, we found that seed germination was slower in mixtures compared with monocultures, implying inhibitory interactions between species. Seed germination is known to be affected by the presence of other species, via a number of interspecific seed‐to‐seed allelopathic effects (Friedman & Waller, 1983). In fact, it is well established that cereals possess allelopathic abilities (Weston & Duke, 2003; Zeng et al., 2008). For example, extracts from wheat seeds and other cereal tissues inhibit the germination and seedling growth of weed species (Fernández‐Aparicio et al., 2013; Ghafarbi et al., 2012) and wheat seedlings can inhibit the root growth of ryegrass (*Lolium rigidum*) (Wu et al., 2000). Variation in allelopathy within and between species is large and has been linked to competitive ability (Bertholdsson, 2004; Worthington & Reberg‐Horton, 2013; Wu et al., 2000). Likely mechanisms include the inhibitory effects of various chemical groups, usually secondary plant metabolites, including phenolics, flavonoids, terpenoids, and alkaloids (Gniazdowska & Bogatek, 2005).

More broadly, non‐crop examples of allelopathy effects on germination have been shown for the herbaceous perennial *Ligularia virgaurea*, which caused longer germination times and lower root growth rates, and in some cases lower germination rates, in four Tibetan grasses, with smaller‐seeded species seeming to be more sensitive to allelopathic impacts (Zhang et al., 2011). Similarly, the germination, emergence, and seedling root length of musk thistle (*Carduus acanthoides*) were inhibited by the presence of seeds of narrowleaf birdsfoot trefoil (*Lotus tenuis*) (Laterra & Bazzalo, 1999). While these experiments do not perfectly reflect our experimental situation, as we have seeds germinating on soil, in isolation from mature plants, it is possible that we could be seeing allelopathic effects from the roots of germinated seedlings on seeds that have not yet germinated.

In addition to negative effects between species, such as allelopathy, there can also be positive effects. The phenomenon of “overyielding” is well known, occurring when biomass or seed yield is greater for species in mixtures than would be predicted from the species growing in monocultures, both in natural and agricultural systems (Hector et al., 2002; Li et al., 2014). This overyielding may be due to niche differentiation of the species in the mixture, or direct or indirect benefits from one species to another, called facilitation (Li et al., 2014). Strong indications of facilitation were shown when the crop progenitors *H. vulgare* subsp. *spontaneum* and *T. monococcum* subsp. *aegilopoides* were grown in a mixture with the non‐progenitor *T. caput‐medusae*, with all species producing higher biomass than when in monocultures. While it is difficult to speculate on specific mechanisms involved in this study, and whether differences in overyielding exist between crop progenitors and other wild grasses, this is more evidence of the importance of studying plant growth under competition, and not only as individuals.

### 4.4 Seedling survival and biomass under competition

Individual plant survival is closely linked to germination and in one experiment comparing 47 grassland species probability of emergence was the best predictor of cumulative seedling survival (the proportion of sown seeds resulting in survived seedlings) (Larson et al., 2015). This was regardless of watering treatments and the probability of seedling survival (the proportion of established seedlings surviving to the onset of dormancy) (Larson et al., 2015). In our study, there was no difference in the likelihood of survival between crop progenitors and other wild species, indicating that any disparity in competitive ability between progenitors and wild species was due to germination rate and seedling size, rather than plant mortality.

Perhaps, the simplest way of quantifying competitive outcomes between two species is to compare the biomass produced by these species when grown together in a mixture. In this situation, crop progenitors produced a larger biomass than the other wild species, and this was amplified when *A. sterilis* and *S. vavilovii* were also included in the progenitor group. This contrasts with the situation for these same species at maturity (after grain production), where there were no differences in aboveground biomass between crop progenitors and other wild species, either when plants were grown as individuals or under monospecific competition (Preece et al., 2015, 2018). Since neither of these earlier studies tested species mixtures, this shows the importance of the current study in highlighting the changes in plant traits that can occur when there is interspecific competition.

Asymmetric competition between species early during the establishment phase, as we show here between *H. spontaneum* and the other wild grasses, can persist and create large differences in longer term species success (Freckleton & Watkinson, 2001). As with our results for germination, we found evidence of variation in seed mass being especially important for determining seedling competitive ability. This would have reinforced the selection for larger seed size, a trait in which progenitors already had an advantage. It is also clear that there are large differences between species. The barley progenitor was a much stronger competitor than the einkorn wheat progenitor, which means that generalizations based on only two species should be approached with caution. The inclusion of progenitors of the secondary crops oat and rye in our analyses (as progenitors) tended to produce similar results or to enhance the difference between progenitors and other wild species. This may indicate that comparable processes of selection acted on primary and secondary cereal crop progenitors. Together, these species share a suite of functional traits that may have given them a competitive advantage over other wild species, and this implies that similar mechanisms of selection may have applied to the initial domestication of the primary “founder” crops and the later domestication of oat and rye.

### 4.5 Conclusions

With this study, we have made a first step toward understanding how cereal crop progenitors may have gained a competitive advantage over other wild grasses during the beginnings of domestication. However, the complex interactions between plants, soil microbes, animals, and climate, as well as human socio‐economic factors, place limitations on determining how the plant species in this study would have experienced and responded to competition at this time. Fertile Crescent cereal crop progenitors are generally very similar to the other western Asian grasses in these experiments, but there are a few key traits in which they differ. In particular, in mixed species stands, larger‐seeded cereal crop progenitors tended to have higher germination success and reach a larger size than other wild grasses. Additionally, increases in seed size are particularly advantageous for crop progenitors, because their larger seeds gave additional benefits for fitness and competitive success through higher seed germination success and faster germination. Thus, selection under competition for larger seeded individuals would have been stronger in the crop progenitors than in the other wild species. This combination of traits would have led to larger‐seeded crop progenitors being able to out‐compete other wild grasses for light and nutrients at the seedling stage, and thus promote the persistence of these species in areas of human settlement, or in early cultivated plots. Thus, the close links between seed size and competitive ability offer a way that crop progenitors may have amplified their advantage over other grasses during the origins of agriculture.

## CONFLICT OF INTEREST

The authors declare that there are no conflicts of interest.

## AUTHOR CONTRIBUTIONS

**Catherine Preece:** Data curation (lead); Formal analysis (lead); Methodology (equal); Writing‐original draft (lead). **Glynis Jones:** Conceptualization (equal); Funding acquisition (equal); Supervision (equal); Writing‐review & editing (equal). **Mark Rees:** Conceptualization (equal); Formal analysis (supporting); Funding acquisition (equal); Supervision (equal); Writing‐review & editing (equal). **Colin Osborne:** Conceptualization (equal); Funding acquisition (equal); Methodology (equal); Project administration (equal); Supervision (equal); Writing‐review & editing (equal).

## Supporting information

Supplementary Material

## Data Availability

Trait data from the growth and competition experiments are available at Figshare, with https://doi.org/10.6084/m9.figshare.13487637.
