# Supplementary material for: Fertile Crescent crop progenitors gained a competitive advantage from large seedlings
Source: Ecol Evol. 2021 Mar 4;11(7):3300–12. doi: 10.1002/ece3.7282 (PMC8019021; doi:10.1002/ece3.7282)

Supplementary Online Information for Fertile Crescent crop progenitors gained a competitive advantage from large seedlings

Catherine Preece, Glynis Jones, Mark Rees, Colin P. Osborne

Table S1. List of grass accessions used, including domestication status, accession number on the GRIN database, country of origin and mean initial seed mass in mg (±1SE).

Species	Status	Accession numbers	Country of origin	Mean seed mass mg (± 1SE)	Used in growth experiment	Used in competition experiment
Aegilops speltoides Tausch	Wild	PI 170204	Turkey	6.8 ± 0.27	Yes	Yes
		PI 487231	Syria	6.3 ± 0.57	Yes	Yes
Aegilops tauschii Coss.	Wild	PI 486275	Turkey	8.7 ± 0.37	Yes	No
		PI 511370	Iran	16.1 ± 1.08	Yes	No
Avena fatua L.	Wild	PI 173584	Turkey	10.9 ± 0.60	Yes	No
		PI 432457	USA	10.5 ± 0.87	Yes	No
Avena sterilis L.	Wild	PI 309527	Israel	11.5 ± 0.72	Yes	No
		PI 220372	Afghanistan	16.1 ± 0.72	Yes	No
		PI 134251	Afghanistan	22.3 ± 1.09	No	Yes
		PI 309424	Israel	13.6 ± 1.31	No	Yes

		PI 326955	Israel	11.2 ± 0.59	No	Yes
Bromus brachystachys Hornung	Wild	PI 220582	Afghanistan	2.4 ± 0.03	Yes	Yes
Eremopyrum bonaepartis (Spreng.) Nevski	Wild	PI 227345	Iran	3.1 ± 0.10	Yes	Yes
		PI 227343	Iran	1.8 ± 0.07	Yes	Yes
Eremopyrum orientale (L.) Jaub. & Spach	Wild	PI 229425	Iran	3.1 ± 0.08	Yes	No
		PI 219963	Iran	2.8 ± 0.06	Yes	No
Hordeum marinum subsp. *gussoneanum* (Parl.) Thell.	Wild	PI 401358	Iran	2.9 ± 0.21	Yes	No
Hordeum vulgare L. subsp. *spontaneum* (K. Koch) Thell.	Progenitor	PI 282656	Israel	12.9 ± 0.79	Yes	Yes
		PI 466126	Syria	28.8 ± 0.88	Yes	Yes
Phalaris paradoxa L.	Wild	PI 202684	Turkey	2.2 ± 0.29	Yes	No
		PI 380964	Iran	1.8 ± 0.04	Yes	No
Secale strictum (C. Presl) C. Presl	Wild	PI 383756	Turkey	5.5 ± 0.22	Yes	No
Secale vavilovii Grossh.	Secondary progenitor	PI 284842	Hungary	28.8 ± 0.71	Yes	Yes
		PI 573649	Afghanistan	21.7 ± 0.63	Yes	Yes
Taeniatherum caput-medusae (L.) Nevski	Wild	PI 577710	Turkey	7.8 ± 1.90	Yes	Yes
		PI 577709	Turkey	4.1 ± 0.11	No	Yes

Triticum monococcum L. subsp. *aegilopoides* (Link) Thell.	Progenitor	PI 352503	Iran	14.2 ± 0.66	Yes	No
		PI 527697	Canada	12.4 ± 0.99	Yes	No
		PI 245726	Turkey	16.1 ± 2.25	No	Yes
		PI 427452	Turkey	14.5 ± 1.39	No	Yes
Triticum turgidum L. subsp. *dicoccoides* (Körn. ex Asch. & Graebn.) Thell.	Progenitor	PI 352324	Lebanon	32.1 ± 1.29	Yes	No
		PI 428022	Turkey	23.2 ± 0.98	No	Yes
		PI 300989	Israel	27.3 ± 1.04	No	Yes

Table S2. Species used in experiments 1 and 2 of the growth experiment.

Species	Experiment 1	Experiment 2
Aegilops speltoides Tausch	X	X
Aegilops tauschii Coss.	X	X
Avena fatua L.	X	X
Avena sterilis L.	X	X
Bromus brachystachys Hornung	X	X
Eremopyrum bonaepartis (Spreng.) Nevski	X	X
Eremopyrum orientale (L.) Jaub. & Spach	X	X
Hordeum marinum subsp. *gussoneanum* (Parl.) Thell.	X	
Hordeum vulgare L. subsp. *spontaneum* K. Koch) Thell.		X
Phalaris paradoxa L.	X	X
Secale strictum (C. Presl) C. Presl	X	
Secale vavilovii Grossh.	X	X
Taeniatherum caput-medusae (L.) Nevski	X	
Triticum monococcum L. subsp. *aegilopoides* (Link)	X	X
Triticum turgidum L. subsp. *dicoccoides* (Körn. ex Asch. & Graebn.) Thell.	X	

Table S3. Species used in the competition experiment showing the nine species grown as monocultures and the eight combinations of wild-progenitor pairs.

Monocultures	Mixtures		
Aegilops speltoides (wild)	*Aegilops speltoides* (wild)	and	*Hordeum vulgare* subsp. *spontaneum* (progenitor)
Avena sterilis (secondary progenitor)	*Bromus brachystachys* (wild)	and	*Hordeum vulgare* subsp. *spontaneum* (progenitor)
Bromus brachystachys (wild)	*Erempyrum orientale* (wild)	and	*Hordeum vulgare* L. subsp. *spontaneum* (progenitor)
Eremopyrum orientale (wild)	*Taeniatherum caput-medusae* (wild)	and	*Hordeum vulgare* subsp. *spontaneum* (progenitor)
Hordeum vulgare subsp. *spontaneum* (primary progenitor)	*Aegilops speltoides* (wild)	and	*Triticum monococcum* subsp. *aegilopoides* (progenitor)
Secale vavilovii (secondary progenitor)	*Bromus brachystachys* (wild)	and	*Triticum monococcum* subsp. *aegilopoides* (progenitor)
Taeniatherum caput-medusae (wild)	*Eremopyrum orientale* (wild)	and	*Triticum monococcum* subsp. *aegilopoides* (progenitor)
Triticum monococcum subsp. *aegilopoides* (primary progenitor)	*Taeniatherum caput-medusae* (wild)	and	*Triticum monococcum* subsp. *aegilopoides* (progenitor)
Triticum turgidum subsp. *dicoccoides* (primary progenitor)			

Fig. S1. Mean natural logged plant biomass of species in the competition experiment (data for monocultures and mixtures pooled together where relevant).

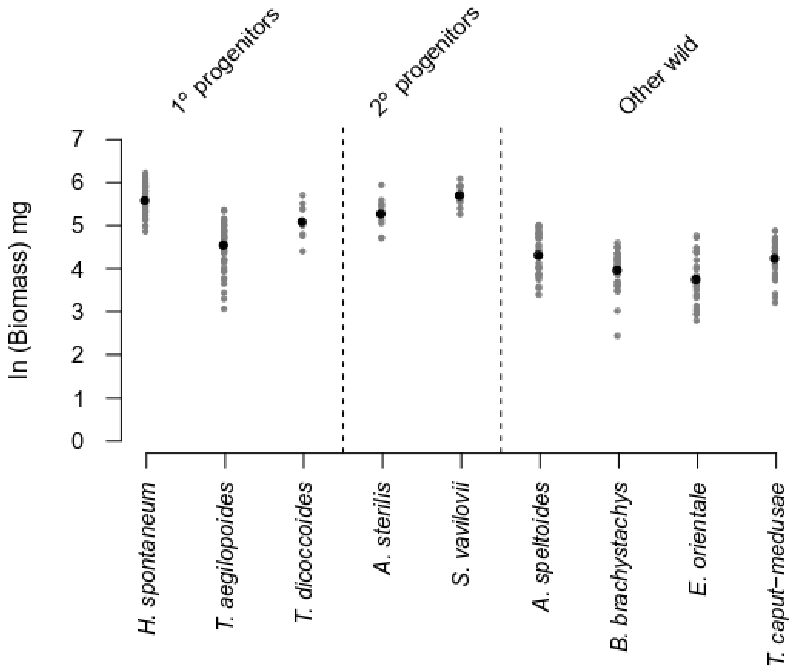

Fig. S2. The relationship between natural logged plant biomass and natural logged individual seed mass ($P < 0.01$). Colours of points show the combinations of domestication status (prog = progenitor) and stand type (mix = mixture, and mono = monoculture).

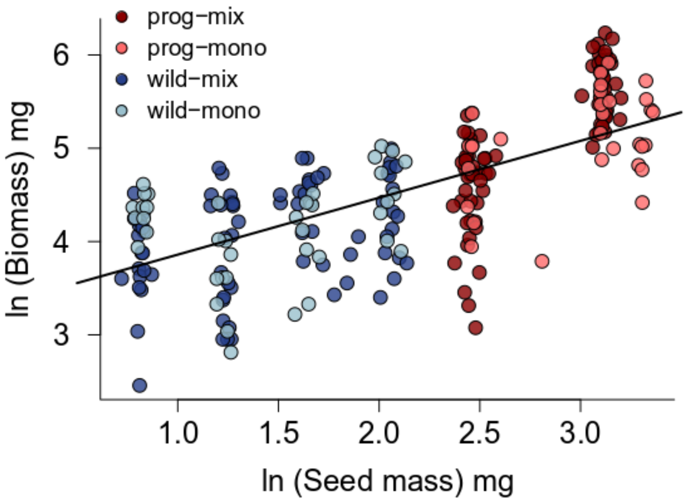

Supplement: Supplementary file 1 — Supplementary Material [file ECE3-11-3300-s001.pdf]
